# Ergonomic Risk Factors of Teleworking in Ecuador during the COVID-19 Pandemic: A Cross-Sectional Study

**DOI:** 10.3390/ijerph18105063

**Published:** 2021-05-11

**Authors:** César Larrea-Araujo, José Ayala-Granja, Andrea Vinueza-Cabezas, Patricia Acosta-Vargas

**Affiliations:** 1 Facultad de Ingeniería y Ciencias Aplicadas, Carrera de Ingeniería Industrial, Universidad de Las Américas, Quito 170125, Ecuador; cesar.larrea@udla.edu.ec (C.L.-A.); jose.ayala.granja@udla.edu.ec (J.A.-G.); 2 Escuela de Psicología, Universidad de Las Américas, Quito 170125, Ecuador; andrea.vinueza@udla.edu.ec; 3 Intelligent and Interactive Systems Laboratory, Universidad de Las Américas, Quito 170125, Ecuador; 4 Department of Software and Computing Systems, University of Alicante, 03690 Alicante, Spain

**Keywords:** ergonomics, COVID-19, human factors, safety, teleworking

## Abstract

Due to the COVID-19 global pandemic, guidelines for people’s confinement have been implemented to prevent the disease’s spread. As a result of this, companies have implemented teleworking as an emerging way to work from home using information technology. This cross-sectional study was conducted in Ecuador, with a sample of 204 teleworkers in the city of Quito. The results show that the teleworkers rearranged their bedrooms to carry out their activities. The respondents in each age group stated they did not perceive more significant ailments than those experienced before beginning teleworking. The relationships between the variables were analyzed utilizing the Chi-Square test and Fisher’s exact test, finding a relationship between neck ailments and age of *p* = 0.031 * and between arm/forearm ailments of *p* = 0.032 *. This study contributes to a greater understanding of the ergonomic situation of the teleworkers and provides us with information to mitigate the ergonomic risks to which they are exposed.

## 1. Introduction

In January 2020, the COVID-19 epidemic was determined to be a global pandemic due to the rapid spread and high level of infectivity [1,2]. A state of emergency was declared in Ecuador on 11 March 2020. On 12 March, guidelines for telework to be used during the health emergency period were published [1,3].

Confinement due to the SARS-CoV-2 virus has caused radical changes in global society [4], in the forms of social contact and in the way of working, giving way to telework, as companies closed according to the regulations of each country and sent their workers to work from home, giving way to telework [1,5,6]. According to Internet World Stats [7], by the end of 2020, the number of internet users grew; approximately 5 billion individuals use this service, representing 63.2% of the world’s population.

The “new normal” is a term used by several countries in the world, particularly by the government of Spain [8], by world leaders of the World Health Organization [9], and the Prime Minister of Canada [10]. They coincide in pointing out that its determining factors include the social distancing of humanity, the use of masks, the limitation of the concentration of people in closed places, and the establishment of rigid aseptic norms, among other things.

From the organizational and productive perspective, public and private companies have found it necessary to ensure the lowest economic and service impact through a practical proposal to their workers to undertake their activities from home [1,11]. However, in most cases, this new work environment does not have the optimal infrastructure that meets the elementary standards of comfort for the productive activity entrusted to it [2,12].

Teleworking originated in the 1970s due to the oil crisis, wherein some employees performed their work remotely. It became evident for the first time that this modality could provide flexibility in the development of work that can benefit organizations and individuals [9,13]. Telework refers to work activities performed outside the company’s premises, and with the use of information and communication technology [2,14,15].

The Council of the International Ergonomics Association states: “ergonomics is a multidisciplinary scientific discipline that studies the relationships between people, the activities they perform, and the elements of the system in which they are immersed, to reduce the physical, mental and psychological loads of the individual and adapting products, systems, workstations, and environments to the characteristics, limitations, and needs of their users; seeking to optimize their efficiency, safety, comfort and overall system performance” [16].

On the other hand, occupational exposure to ergonomic risk factors is defined as “occupational exposure to one or more of the following factors: forceful exertion, demanding posture, repetitive motion, hand-arm vibration, kneeling or squatting, lifting and climbing” [17].

One of the objectives of ergonomic design is to optimize workload, avoid adverse effects on the worker’s health, and contribute to the productivity and efficiency of organizations [18]. When we talk about occupational risks, the main one that has been detected is the ergonomic risk related to the impact of environmental pollution arising from the use of information and communication technologies [15].

Furthermore, it is essential to establish work conditions [16], such as fixed computers, laptops, tablets, and phones connected to the network. Additionally, the workspace must consider the desk, chair, and the area of the house acting as the improvised office, which determine the teleworker’s capacity as a user of a video display terminal (VDT). A VDT user is considered a worker who uses a VDT for part of his or her time [15]. The legislation in different countries defines a VDT user as a worker who uses this equipment for one hour or more, for example, in Sweden [16]. In Spain, a VDT user spends 4 h per day or 20 h per week with the VDT [14], which has been taken as a basis in many countries. Some health consequences due to the intensive use of VDT are recognized: visual fatigue, headaches, stress, and neck, lumbar, dorsal, arm, forearm, and hand pain, among others [19]. These conditions are related to ergonomic and psychosocial risk factors.

One of the consequences of the ergonomic risk factors mentioned is eye problems [16]. These can occur in workers due to the excessive use of screens at work and outside of work, inadequate lighting, an unstable or defective screen, the poor orientation of the screen or keyboard, and lack of work breaks, which can lead to visual fatigue [16,20,21]. Another important consequence is musculoskeletal disorders. According to the European Agency for Safety and Health at Work, the most frequently occurring risk factors are repetitive hand or arm movements (65% of respondents) and prolonged sitting posture (59%) [22].

As work with a VDT is mainly performed in a sedentary posture, musculoskeletal disorders occur in many workers. This is also influenced by the organization and design of the workstation, and the repetitive movements at the hand, wrist, and finger level [16,19,20]. Due to the wrong postures, the musculoskeletal stress can be prolonged, maintained, or forced [23]. The workstation’s design refers to aspects related to lighting, temperature, noise, vibrations, and the presence of radiation [20].

Similarly, it considers the furniture, chairs, desks [19], screens, keyboards, or poorly placed peripherals, which contribute to users adopting inadequate postures and the arising of cervical, dorsal, lumbar, shoulder, neck, arm, wrist, and hand pain. Additionally, no less critical is the lack of information or training for workers [16].

A systematic review showed that telework is a tool that facilitates the work–life balance, helping to improve the well-being of employees; however, it was emphasized that companies must adopt specific strategies to influence the telework experience [24] positively. Additionally, a study [18] analyzed the relationship between digital technologies in logistics, ergonomics, and work intensification. It was found that the opportunities to replace these repetitive, stressful, and ergonomically disadvantageous tasks did not occur, despite the application of digital technologies in the logistics arena such that the possible positive ergonomic effects could be eliminated.

In turn, another study [17] proved that confinement resulted in positive lifestyle changes and decreased musculoskeletal pain in university professors in Spain. Besides this, it has been confirmed that there is a high prevalence of musculoskeletal disorders, mainly in the dorsal–lumbar spine and neck, and there is an association of these disorders with ergonomic risk factors, prolonged posture and long working hours [24].

Finally, a systematic review and meta-analysis [25] suggested that occupational exposure to ergonomic risk factors can cause osteoarthritis and other musculoskeletal diseases (excluding back and neck pain). It was found that there is a 0.76 prevalence of ergonomic risk factors [25], with no statistically significant differences in exposure by genre, but differences by age group, occupation, and country.

This study answers the following research questions: (1) How are workers prepared to face work activities from home? (2) Are organizations prepared to meet the furniture needs of workers in their new physical workspace? (3) Do organizations respect business hours? (4) Does telework during the pandemic lead to musculoskeletal problems for workers?

## 2. Materials and Methods

### 2.1. Study Setting

A cross-sectional design was used in this study. A descriptive analysis of the data obtained from a survey applied to graduates and professionals of the Universidad de Las Américas in Quito—Ecuador was carried out to confirm the adequate application of ergonomic factors relevant to teleworking. A descriptive statistical analysis was performed in which the frequencies of the categorical variables were analyzed to identify possible relationships. This study applied the Chi-square test, and when the counts were more significant than 5, we considered the Fisher test. Additionally, the IBM–Statistical Package for the Social Sciences (SPSS) software, version 25 (IBM, Armonk, NY, USA), and Microsoft Excel were used for this analysis.

### 2.2. Participants and Procedure

In the sample taken from the city of Quito, we applied a formula to calculate the size of an infinite population because the size of the universe is unknown, with Equation (1).

(1) $$n=\frac{Z^{2}*p*q}{e^{2}}\;$$

The sample includes a confidence level (*Z*) of 95% and a margin of error (*e*) of 7% (suggested sample of 196 surveys), referring to the population dedicated to teleworking (*p* value = 50% and *q* value = 50%). This study applied 204 surveys to professional graduates of Industrial Production Engineering and Organizational Psychology from the Universidad de Las Américas of Ecuador, who are currently active workers, provide technical–administrative services, and are within the circle of professional contacts of the authors of this investigation.

The survey was developed using Google Forms and distributed by sending the link via emails and messages through the WhatsApp platform. The participants could choose whether they wished to participate in the proposed study freely and voluntarily; the research and academic purpose of the study were emphasized. The collection of information did not compromise the integrity, and the confidentiality and anonymity of the responses were guaranteed. The survey was active for ten days between December 17th, 2020 and January 27th, 2021. The response tabulation was developed using Excel spreadsheets; the dataset is available for replication in the Mendeley repository [26].

### 2.3. Measures

The survey was structured into four fields: (1) demographic data, (2) temporal ergonomics, (3) ergonomics in the workplace, and (4) health effects. It included 17 items with single-choice and multiple-choice response scales. The questions included in the survey were an adaptation of the “Nordic Questionnaire”, elaborated and proposed to the International Scientific Community in 1987 [27], which is widely used in musculoskeletal symptomatology amongst working populations and for different anatomical locations. The questions included in the field “Health effects (health consequences)”, related to the discomfort experienced in the “last few weeks”, correspond to an adaptation of questions 1, 2, and 10 of the Nordic, or Kuorinca, questionnaire (See Table A1). The survey included the variables of age, gender, and those that were directly related to the conditions of furniture and the environment in the teleworking space, as proposed by the authors.

#### 2.3.1. Demographic Data

As demographic data, information was collected on the genders and ages of the participants to analyze the variables. Regarding age, the survey assumed ranges with intervals of ten years, starting from the age at which people have autonomy and can make the decision to work [28], i.e., from 18 years of age, advancing in ranges of ten years.

#### 2.3.2. Temporal Ergonomics

Temporal ergonomics [29] dictates studies of workers’ well-being, as regards working times, depending on the type of work and organization, and seeks to mitigate worker’s physical and mental fatigue. It involves three parameters:

- Time in Telework—this determines the degree of involvement and the time each participant is immersed in telework, without the supervision of the organization’s Occupational Health and Safety personnel;
- Working time with information and communication technologies ICT—net activity in which the worker is exclusively immersed in contact with stakeholders using information technologies, particularly concerning the flow of valid information to achieve organizational objectives;
- Working time at home without the use of information and communication technologies ICTs—activities not focused on work-related issues and the organization of administrative work. These actions, productive and intellectual, are detrimental to the worker’s health since the teleworker remains at home to perform the work with the aggravating factor of intellectual effort and the pressure to complete the task in each time.

#### 2.3.3. Ergonomics in the workplace

- This considers environmental ergonomics—noise, lighting, temperature, place where the work is performed, furniture used, and the duration of the telework.
- Home area for teleworking—specific place in the home that is set aside for teleworking.
- Environmental ergonomics—the existing environment in the work area is essential in the normal performance of activities, especially as regards the perception of comfort in the workplace; thus, it includes questions related to the worker’s comfort, noise, lighting, and temperature.
- Location of the workstation as regards natural light—refers to the criteria for the location of windows that allow natural light to enter during the workday; they should be located on the sides of the worker, as this avoids possible light reflections that would damage a person’s eyesight when in front of a computer data display screen [16,20]. This element is applied due to the geographic location of Quito, Ecuador, which has natural light 12 h a day throughout the year.
- Work furniture—use of ergonomic chairs and furniture suitable for the teleworking activity and devices used, leading to discomfort in workers.

#### 2.3.4. Effects on Health

- This refers to musculoskeletal symptoms and is determined via discomfort in the neck, shoulder, lumbar back, arm–forearm, wrist–hand, and lower extremities; the relationships of these ailments are detailed in the following.
- Lumbar pain—lumbar spine ailments are attributed to inadequate or prolonged sedentary postures.
- Neck pain—neck disorders are caused by defects in the location of the screen (too high or too low), or the use of smartphones or tablets, causing cervicalgia, which could lead to subsequent problems of dizziness, headaches, and other problems for the spine [16].
- Wrist and hand pain—the discomfort that workers have in the wrist and/or hand is related to repetitive movements, typical of typing on computers and other devices, which can trigger various problems such as carpal tunnel syndrome or various types of tendinitis, including the inflammation of tendons that go to the thumb due to smartphone use [16].
- Discomfort in the forearm, elbow and arm—this has not been directly related to VDT work, although recently, it has been suggested that epicondylitis could be caused by keyboard and mouse use [16,30].
- Shoulder ailments—shoulder complaints may be related to poor posture caused by poor desk design or poor keyboard placement, as well as digitization or poor screen placement [16].
- Lower extremity discomfort—it would also be beneficial to reduce the discomfort in the lower extremities due to prolonged sedentary posture.

## 3. Results

- The data tabulated for the questions posed to the survey participants present the results related to ergonomics and telework.

### 3.1. Frequencies of the Variables Studied

- This study presents the results obtained from the descriptive analysis of the frequencies of responses obtained for each category. From the 204 valid surveys carried out, the data obtained were as follows: 91 correspond to the male gender, representing 45% of the total, and 113 respondents were female, at 55%. The most significant group of participants within the selected sample was the 115 people aged between 25 and 34, representing 56% of the total. This study revealed that 91% of workers (See Table A1) consider themselves users of TDV because they spend more than four hours a day in front of the computer [20].

This research shows that teleworkers use various places in the house to work; 36% do it in the bedroom, 25% in the study, 20% in the dining room, and 16% in the living room, confirming that they do not have an adequate workspace given the circumstances of COVID-19.

On the other hand, 81% of the sample use more than one device, such as desktop computers, laptops, tablets, and even cell phones, to carry out their activities. In total, 68% answered that they would be willing to continue teleworking after the pandemic occasionally. Additionally, the principal health effects were in the lumbar area of the back (57.3%) and in the neck area (58.8%).

### 3.2. Relationships between the Workplace, Ailments, and the Use of an Ergonomic Chair

As regards the relationships between the study variables, we used cross-tabulations to analyze workplaces and ailments. Table 1 shows the results of the perception of ailments in relation to the place used during teleworking. The highest incidence of back pain was in the lumbar region (68.5%) and the neck (67.1%) when working in the bedroom.

Assessing the health effects and the place used to perform teleworking revealed that when using a studio with an ergonomic chair as the workspace, more than 50% of the participants presented no more significant ailments than when working in offices. On the other hand, the worst workstation arrangement was in the bedroom, without using an ergonomic chair, because neck and back ailments were shown to increase. Likewise, teleworkers who performed work activities in the bedroom or the dining room without an ergonomic chair displayed hand or wrist discomfort at a rate of 42.2%. Of the workers who use a study to carry out their work activities with an ergonomic chair, 44% perceived discomfort at the lumbar level of the back, compared to 55.6% of those who did not have an ergonomic chair.

On the other hand, more than 50% of teleworkers who worked in the bedroom or the dining room suffered afflictions in the back and neck, regardless of the type of chair they used (See Table A2).

### 3.3. Results of Complaints Concerning the Age Groups

Table 2 shows a summary of the 204 participants organized by age; most participants from all age groups presented with more complaints than usual in the back (57.4%) and neck (58.8%), except for the group aged between 45 and 54 years. As for the other ailments, the respondents in each age group stated that they did not perceive more significant ailments than before commencing teleworking. Additionally, the relationships between the variables were analyzed utilizing the Chi-Square test, and for a value less than 5, the Fisher’s exact test was used, finding a relationship between neck ailments and age of *p* = 0.031 * and between arm/forearm ailments of *p* = 0.032 *.

### 3.4. Results on Telework Continuity Concerning Age

This study considered it essential to analyze the motivation of the participants to continue teleworking according to age. Table 3 shows that the group between 18 and 24 of age years had a preference of 82.4%. Of the participants between 25 and 34 years old, 70.4% preferred teleworking. In turn, 51.4% of employees between 35 and 44 years old chose the telework modality. People between 45 and 54 years old showed a 53.8% preference for this modality. Finally, 60% of participants over 55 years old prefer to work occasionally in teleworking.

The Fisher’s exact test was applied to identify a relationship between the variables of interest in teleworking and age with a value of *p* = 0.032 *.

## 4. Discussion

In this research, the factors that influence teleworking were analyzed. It was evident that teleworkers have arranged their homes to perform work activities. This study revealed the prevalence of problems in the physical environment, such as excess noise, lack of lighting, and excess heat. These factors cause increased musculoskeletal problems due to the lack of adequate furniture to carry out telework activities. This study helps us to understand the ergonomic situation of teleworkers due to COVID-19, and provides information to help mitigate the ergonomic risks to which they are exposed.

In this study, it was determined by data related to temporary ergonomics that most of the respondents have worked more than 4 h per day for seven months, which means that workers are subjected to the probability of suffering from various health alterations in the future due to the intensive use of the video display terminal [16,17,20].

The participants found it necessary to improvise a workplace environment from home due to the COVID-19 pandemic [2], with no particularly encouraging results other than the obligation to guarantee greater comfort in the workplace [31,32]. This study revealed that most of the workers carry out their activities in spaces designed for home life, such as the dining room, the living room, or the bedroom, and almost half of these environments do not have adequate furniture, which leads to the conclusion that the design of these teleworking places is not ideal from an ergonomic point of view [16,19,20].

After seven months of the pandemic, more than half of the participants experienced a sensation of tension in the back, lower back, and neck. About a third felt the same in the arm, forearm, hand, wrist, and shoulders. These results are consistent with studies conducted by international occupational health and safety organizations on the use of video display terminals [17,19,20].

This study made it possible to establish some relationships between variables that aim to verify pre-established ideas around minimizing the impact of musculoskeletal disorders.

The first analysis helped us understand the possible effects on people’s health depending on their work [20]. The disease with the highest incidence was related to the neck and back at the lumbar level; the most affected group was the participants who carried out their activities from the bedroom, with more than two-thirds of these presenting discomfort, compared to those who worked in an office. Some of the health afflictions include cervical pain, back pain, herniated discs, sciatica, disc protrusion, and lumbago.

This study confirmed the importance of properly designing the workstation and providing postural education in the management of the video display terminal [19].

The design of workstations in a sitting position considers the ergonomic chair as an essential element [20]. An ergonomic chair adapts the workspace to the body’s dimensions, and provides stability, freedom of movement, and the ability to adopt a proper posture. From the analysis of the survey participants’ perceptions of the workplace and the use of an ergonomic chair in relation to discomfort in various parts of the body, it is evident that it is more appropriate to work in an office. The respondents stated that they did not perceive significant ailments when they used an ergonomic chair in a place intended for work, such as an office, while considering that the worst place to carry out their work activity is the bedroom. Likewise, they affirmed that the lack of an ergonomic chair produces significant discomfort at the lumbar level of the back and in the neck in all its variants. This study further revealed that most teleworkers who do their work in the bedroom or dining room experience hand or wrist discomfort.

The survey’s results determine that 58.8% of the workers claimed to have pain or discomfort in the neck when using the company’s facilities, where they carried out their usual work. The age range in which this ailment had the most significant impact was between 25 and 34 years, at 32.4% of workers.

On the other hand, this study revealed that the usual workplace is not ergonomically suitable for teleworkers, as it does not meet acceptable technical conditions [16,20]. It is essential to implement equipment that improves worker comfort and avoids musculoskeletal discomfort [16].

In cases where the worker has a desktop computer, the monitor should be raised such that the eyes are level with the top edge of the screen. For laptop users, the authors suggest using an additional keyboard and elevating the device above a desk. This study revealed that 67% of users who work in the bedroom use a laptop computer and work while lying in bed, so they experience neck discomfort.

The age of the respondents and their back problems depend on the type of furniture they use in their makeshift jobs. This study showed that more than half of the workers reported lumbar problems (57.4%), and that the postures adopted in the workplace at home are not adequate; in particular, those aged 25 to 34 were the most affected (31.4%). This study suggests correcting the sedentary posture, emphasizing support of the lumbar area, together with the postural education of the worker in order to minimize low back pain [20].

Besides this, as regards arm and forearm ailments, age is connected to the posture adopted by the worker in front of the computer; 35.3% of all workers reported some ailment or discomfort in their upper extremities, which is about one in three teleworkers. It was observed that the highest percentage of affliction was in workers between 25 and 34 years old, at 19.1%. These ailments are related to the position and height of the work surface and the keyboard and mouse layout [20]; 67.6% of all workers surveyed (two out of three workers) said they would like to distribute work time between the office and at home, and 13.2% would prefer not to telework at all.

The continuation of telework across age groups commits organizations to collaborate in the technical adaptation of the workstations, such as providing adequate furniture for each electronic device, ensuring the timely delivery of information on good teleworking practices, inspecting workstations to avoid health effects in the medium and long term, controlling time flexibility, and maintaining awareness of work breaks. Finally, it is recommended that participants perform physical activities to mitigate the sedentary impact of teleworking [17].

The aspects of this research that we have identified as a limitation are related to the sample, because offers little representation of various age groups. In future research, it is recommended to include more people from the 45 years and older age group. Another limitation identified is the period of data collection; it is advisable to develop longitudinal research to verify whether the elements that create ergonomic risks to workers’ health are maintained over time.

## 5. Conclusions

The data obtained from the survey are focused on ergonomics, considering the physical space, the environment, and the time dedicated to the tasks of teleworkers. The study revealed that the ergonomic conditions currently found in each home are unsuitable for teleworking due to the improvisational nature of the workplace, the need for the organization to remain competitively active, and the low probability that the organization will provide workers with the necessary tools, furniture, and supplies.

Another recurring problem detected in this study was the inappropriate use of the time assigned to work, which affects the musculoskeletal discomfort and even visual fatigue of the workers. It is another element to consider in-depth in future research.

According to this research, most of the people currently carrying out their work in the teleworking modality would be willing to continue with this scheme, so it is necessary to inform the population about the ideal conditions for preparing the workspaces in order to avoid long-term health problems.

The activity of the company’s Occupational Health and Safety personnel implies some appropriate measures, such as the following: (1) Conduct periodic interviews with the worker to provide timely advice based on a questionnaire on occupational health problems. (2) Prepare, maintain, and update information on the participant’s behavior related to the use of the computer, their time in front of it, the implementation of active and passive breaks, and relaxation exercises during teleworking. (3) Ask the organization to provide furniture, tools, supplies, and other necessary implements that facilitate the worker’s well-being. (4) Inform and train workers on the risks inherent in using data display screens and the benefits of proper job design.

Due to the lack of control in teleworking, it has been detected that many workers develop afflictions to their visual systems, with a pathology called “dry eye” that corresponds to discomfort in the eyes due to a lack of lubrication after not blinking regularly; this is a topic the authors suggest for future research.

This article can serve as a reference for studies related to telework from various perspectives, such as the suitability of the telework position, musculoskeletal consequences, prevention mechanisms, and ergonomic risk factors. The study of psychosocial risk, because of its depth, deserves a separate analytical approach that could be developed in future studies; however, some aspects that are not considered by the science of work psychosociology cannot be dissociated, but must necessarily be addressed by temporary ergonomics.

## Figures and Tables

**Table 1:** Perception of ailments by telework location.

Greater than Usual Pain	Bedroom			Study			Dining			Room Living Room			Others		
	*N*	*n*	%	*N*	*n*	%	*N*	*n*	%	*N*	*n*	%	*N*	*n*	%
Back	73	50	68.5	52	26	50.0	41	21	51.2	33	15	45.5	5	5	100.0
Neck	73	49	67.1	52	26	50.0	41	23	56.1	33	17	51.5	5	5	100.0

*N* = total number of workers in each condition, out of 204 respondents. *n* = number of workers with complaints/responses of “Somewhat more than usual” and “Much more than usual.” % = percentage of workers out of the total “*N*” in each condition.

**Table 2:** Ailments according to the age of the respondents.

Pain Greater than Usual		Participants	Back	Neck	Arm/Forearm
Age	18–24	***N***	34	34	34
		***n***	23	22	16
		**%**	67.60	64.70	47.10
	25–34	***N***	115	115	115
		***n***	64	66	39
		**%**	55.70	57.40	33.90
	35–44	***N***	37	37	37
		***n***	21	27	9
		**%**	56.80	73.00	24.30
	45–54	***N***	13	13	13
		***n***	6	2	5
		**%**	46.20	15.40	38.50
	Total	***N***	204	204	204
		***n***	117	120	72
		**%**	57.40	58.80	35.30
Fisher’s exact test		0.751		0.031 *	0.032 *

*N* = total number of workers in each condition, out of 204 respondents. *n* = number of workers with complaints/responses of “Somewhat more than usual” and “Much more than usual”. % = percentage of workers versus total “*N*” in each condition. * *p* < 0.05.

**Table 3:** Interest in teleworking modality related to age.

Options	Age	Results	Percentage of Age Group (%)	Total Percentage (%)
Continue teleworking	18–24	2	5.9	1
	25–34	21	18.3	10.3
	35–44	11	29.7	5.4
	45–54	5	38.5	2.5
	+55	0	0.0	0
Occasional teleworking	18–24	28	82.4	13.7
	25–34	81	70.4	39.7
	35–44	19	51.4	9.3
	45–54	7	53.8	3.4
	+55	3	60.0	1.5
Dislike of teleworking	18–24	4	11.8	2
	25–34	13	11.3	6.4
	35–44	7	18.9	3.4
	45–54	1	7.7	0.5
	+55	2	40.0	1
Total		204		
Fisher’s exact test	0.032 *			

* *p* < 0.05.

## Data Availability

Data available on request.

## Appendix A

**Table A1:** Frequencies of the Variables Studied.

Variables	Question	Response Options	Results (%)
Statistical demographics	Gender	Female	55.0%
		Male	45.0%
		Other	0.0%
	Age group	18–24 years of age	17.0%
		25–34 years of age	56.0%
		35–44 years of age	18.0%
		45–54 years of age	6.0%
		Over 55 years of age	2.0%
Temporary ergonomics	How long have you been teleworking?	Less than one month	7.0%
		From one to three months	11.0%
		From four to six months	12.0%
		More than seven months	69.0%
	How many hours per day do you telework?	Less than 3 h per day	1.0%
		3 to 4 h per day	9.0%
		4 to 5 h per day	8.0%
		More than 5 h a day	81.0%
	For activities that do not involve teleworking (planning, filing, reviewing documents, and others related to your activity), how many hours per day do you use?	Less than 3 h per day	67.0%
		3 to 4 h per day	23.0%
		4 to 5 h per day	6.0%
		More than 5 h a day	4.0%
Ergonomics in the workplace	To perform telework activities at home, what area of your home do you occupy?	Study	25.0%
		Living room	16.0%
		Dining room	20.0%
		Bedroom	36.0%
		Kitchen	1.0%
		Courtyard or other remote space	1.0%
	The natural lighting in your work area (windows) is located	Behind you	16.0%
		In front of you	20.0%
		To the side	43.0%
		Some combination of the above	21.0%
	Do you have any of these problems in your workplace? (you can select several options):	Too much noise	19.0%
		Too much or too little lighting	8.0%
		Too much heat or too much cold	11.0%
		None of the above	33.0%
		Two or more problems	36.0%
	Once the option of returning to regular work is made possible, you would like to:	Continue in telework mode	19.0%
		Occasionally telecommute	68.0%
		You would not like to telework	13.0%
	Do you have furniture to develop the Telework activities?	Yes	54.0%
		No	17.0%
		Some furniture	29.0%
	Is your work chair at least adjustable in height, with a backrest adjustable in-depth and a swivel base with five wheels?	Yes	37.0%
		No	63.0%
	For your teleworking activities do you have (you can select several options):	More than one device	81.0%
		Desktop computer	0.5%
		Laptop	16.5%
		Cellphone	0.5%
		Tablet PC	0.5%
		Only one device	18.0%
Health effects (health consequences)	In the last few weeks, have you experienced back discomfort at the lumbar level?	No, not at all.	14.7%
		No more than usual.	27.9%
		Somewhat more than usual.	44.6%
		Much more than usual.	12.7%
	In recent weeks have you experienced neck discomfort?	No, not at all.	16.7%
		No more than usual.	24.5%
		Somewhat more than usual.	42.2%
		Much more than usual.	16.7%
	In recent weeks have you experienced wrist and/or hand discomfort?	No, not at all.	24.5%
		No more than usual.	33.3%
		Somewhat more than usual.	17.6%
		Much more than usual.	24.5%
	In recent weeks have you experienced discomfort in your arm or forearm?	No, not at all.	33.8%
		No more than usual.	30.9%
		Somewhat more than usual.	20.6%
		Much more than usual.	14.7%
	In recent weeks have you experienced shoulder discomfort?	No, not at all.	43.1%
		No more than usual.	27.0%
		Somewhat more than usual.	14.7%
		Much more than usual.	15.2%
	In the last few weeks, have you experienced discomfort in some parts of your lower extremities?	No, not at all.	47.5%
		No more than usual.	28.9%
		Somewhat more than usual.	14.2%
		Much more than usual.	9.3%

**Table A2:** List of ailments by telework place with or without the use of an ergonomic chair.

**More than Usual Pain WITH Ergonomic Chair**	**Bedroom**			**Study**			**Dining Room**			**Living Room**			**Others**		
	*N*	*n*	%	*N*	*n*	%	*N*	*n*	%	*N*	*n*	%	*N*	*n*	%
**Back**	28	17	60.7	25	11	44.0	11	6	54.5	8	4	50.0	4	4	100
**Neck**	28	15	53.6	25	12	48.0	11	7	63.6	8	4	50.0	4	4	100
**Hand Wrist**	28	14	50.0	25	11	44.0	11	6	54.5	8	1	12.5	4	1	25
**Arm Forearm**	28	11	39.3	25	10	40.0	11	5	45.5	8	2	25.0	4	1	25
**More significant than usual pain WITHOUT ergonomic chair**	**Bedroom**			**Study**			**Dining Room**			**Living Room**			**Others**		
	*N*	*n*	%	*N*	*n*		*N*	*n*		*N*	*n*		*N*	*n*	
**Back**	45	33	73.3	27	15	55.6	30	15	50.0	25	11	44.0	1	1	100
**Neck**	45	34	75.6	27	14	51.9	30	16	53.3	25	13	52.0	1	1	100
**Hand Wrist**	45	19	42.2	27	12	44.4	30	10	33.3	25	11	44.0	1	0	0
**Arm Forearm**	45	16	35.6	27	8	29.6	30	8	26.7	25	10	40.0	1	1	100
